# Supplementary material for: The Interfaces Twitter Elections Dataset: Construction process and characteristics of big social data during the 2022 presidential elections in Brazil
Source: PLoS One. 2025 Feb 3;20(2):e0316626. doi: 10.1371/journal.pone.0316626 (PMC11790155; doi:10.1371/journal.pone.0316626)
Supplement: Attachment — Submitted filename: Response to Reviewers.pdf [file pone.0316626.s001.pdf]

**RESPONSE TO REVIEWERS**

Dear Academic Editor
M.D Gábor Vattay, PhD, DSc
PLOS ONE

We would like to express our gratitude to PLOS ONE journal for the opportunity to review the article "The Interfaces Twitter Elections Dataset: construction process and characteristics of Big Social Data during the 2022 presidential elections in Brazil" and for the valuable comments that have helped us to improve our work.

The authors carefully considered each of the reviewers' and editor's comments and did their best to address each of them in a revised version of the article.

After this careful review, we hope that the manuscript meets the high standards and requirements of the PLOS ONE Journal.

Below, we describe the changes made to the text considering each recommendation. Finally, we clarify that all changes are highlighted in blue in a revised version of the manuscript.

Yours sincerely,

Professor Dr. Sylvia Iasulaitis and authors

## 1. Major Points for Revision

### 1.1 Data Sharing and Volume:

Issue: More details are required about the nature, volume, and mode of data sharing, including any restrictions.
Action: Provide comprehensive information on how the data will be shared, including specifics on the nature and volume of the data, and how researchers can access it. Address any restrictions that may apply, especially concerning the full text of tweets and user information in accordance with Twitter's terms of service (ToS).

Solution: We have included the "Data Availability" section in the article.

### 1.2 Compliance with Twitter's Terms of Service:

Issue: Concern about the legality of sharing full tweets and user information according to Twitter's ToS.
Action: Outline the restrictions from Twitter's ToS and explain how these will be navigated. Ensure that all shared data complies with these terms and provide a clear plan for how this compliance will be maintained.

Solution: We included the section "Conformity with Twitter and Twitter API's Terms of Use and Privacy and Developer Policies" with all the details.

### 1.3 Ethics Statement:

Issue: Lack of an ethics statement addressing the collection and aggregation of potentially sensitive data.
Action: Include a detailed ethics statement. Explain why ethical considerations do or do not apply, and whether a human subject review was conducted or exempted by the authors' institution.

Response:

The Ethics Committee of the authors' institution follows the Brazilian standards applied to scientific research, which are provided in its resolutions available at Reso510.pdf (gov.br) (link) e Frequently Asked Question — (ufscar.br) (link) that:

> Some types of research use methodologies characteristic of the Human and Social Sciences and are exempt from submission to the CEP/Conep System, as provided for in the National Council of Health (CNS) Resolution 510/2016 in its Article 1:
>
> Paragraph one: The following **will not be recorded or evaluated by the CEP/CONEP system**:
> I – public opinion survey with unidentified participants;
> II – research that uses publicly accessible information under the terms of Law 12527 of November 18, 2011;
> III – research that uses information of public domain;
> IV – census research;

V – research using databases with aggregated information, where it is impossible to obtain individual identification.

This research falls within the terms of this resolution and its submission for consideration by the Research Ethics Committee is not required, since:

a. the disclosure of the dataset will not allow the identification of participants, as only the ID will be made available and not individual personal information;
b. the data obtained from Twitter was, at the time of its collection, available for access by researchers, who carried out the collection in accordance with Twitter's ToS;
c. the research uses tweets that were made publicly and can be consulted, used, and reproduced without copyright or intellectual property restrictions, so authorization is not necessary, in accordance with the provisions of the Twitter ToS (detailed in the section "Conformity with Twitter and Twitter API's Terms of Use, and privacy and developer policies" inserted in the article) and the Brazilian legal system on public domain works.

The primary data collected are stored on physical servers on the premises of the research group's home institution, the Federal University of São Carlos. Therefore, the complete dataset is in a secure location, access to which is restricted, controllable, and traceable.

The 153 articles consulted to carry out the literature review, when they mention ethical issues, inform that Twitter data is public and, therefore, exempt from approval by the Research Ethics Committee.

The 13 articles with the largest corpus available in Table 1 of our article also did not go through an Ethics Committee, according to the spreadsheet prepared with this information:

https://docs.google.com/spreadsheets/d/12Ozw7_fhy1RnH-9kI5eczorQVtxCw1fnHMSL5DJHwks/edit?gid=197341791#gid=197341791

| Article Title | Corpus Size | Is there any information about data availability? | Where is the data located? | Is there any information on ethical issues? |
|---|---|---|---|---|

1.4 Data Storage and Repository:

Issue: Details on what will be available on GitHub, including file formats and data preprocessing, are lacking. GitHub storage limitations are also a concern.
Action: Clarify the types of files that will be available on GitHub, their formats, and any preprocessing done. Consider using scientific repositories like Dryad or Zenodo for long-term archival and provide detailed information on how the data will be stored and accessed.

Solution: The data is readily available on the GitHub account of the Interfaces research group for the purpose of evaluation by the PLOS ONE editor and reviewers, together with the requested description. The access link is https://github.com/Interfaces-UFSCAR/ITED-Br?tab=readme-ov-file

1.5 Data Validation:

Issue: Unclear validation of the relevance of content downloaded when querying for candidate nicknames.

Action: Describe the methods used to validate that the downloaded content is relevant, especially for generic candidate nicknames. Provide specific examples or methodologies used to ensure data accuracy.

Solution: We have inserted specific examples in the article.

2. **Minor Comments for Revision**

2.1 Clarify Terminology:

Page 2, Line 41: Replace “data mining” with a more precise term as it usually does not refer to data gathering.

Solution: The request was met, with the term being changed to “data scraping.”

Page 2, Lines 47-49: Clarify the connection between AI and “geographically dispersed” datasets.

Solution: This has been removed.

2.2 Literature Review:

Explicitly mention if the datasets reviewed share tweets or have usage restrictions.

Response: In the “Supporting information” section, all links to the available datasets were inserted, and hyperlinks made in the titles of the articles in Table 1.

A spreadsheet was also created with information related to the 13 largest corpus cited in Table 1 of the article in the systematic review for internal use by the editor and reviewers:

https://docs.google.com/spreadsheets/d/12Ozw7_fhy1RnH-9kI5eczorQVtxCw1fnHMSL5DJHwks/edit?gid=197341791#gid=197341791

2.3 Correct Errors:

a. Page 11, Line 361: Correct the Twitter handle for Ciro Gomes.

Solution: We made the necessary changes.

b. Page 14, Line 476: Indicate if there was an optimization to check if the reference ID had been encountered before.

Solution: We made the necessary changes.

2.4 Enhance Interpretation:

Table 7, Figure 4: Provide additional interpretation, especially for non-Portuguese readers.

Solution: A new table (8) was created with the translation of the content of table 7. In relation to figure 4, an additional explanation was made in the article.

2.5 Please include the following items when submitting your revised manuscript:

A rebuttal letter that responds to each point raised by the academic editor and reviewer(s). You should upload this letter as a separate file labeled 'Response to Reviewers'.

Solution: We made the necessary changes.

2.6 A marked-up copy of your manuscript that highlights changes made to the original version. You should upload this as a separate file labeled 'Revised Manuscript with Track Changes'.

Solution: We made the necessary changes.

2.7 An unmarked version of your revised paper without tracked changes. You should upload this as a separate file labeled 'Manuscript'.

Solution: We made the necessary changes.

2.8 If you would like to make changes to your financial disclosure, please include your updated statement in your cover letter.

We request changes to our financial disclosure in accordance with the following:

This work was funded by FAPESP under grant number 2022/03090-0 under the coordination of Prof. Dr. Sylvia Iasulaitis and BCO number 2023/03704-0 and number 2023/17214-5. This research was supported by the Coordination for the Improvement of Higher Education Personnel - Brazil (CAPES) - Finance Code 001, through the granting of a master's scholarship. (Cover Letter)

2.9 Guidelines for resubmitting your figure files are available below the reviewer comments at the end of this letter.

While revising your submission, please upload your figure files to the Preflight Analysis and Conversion Engine (PACE) digital diagnostic tool, https://pacev2.apexcovantage.com/

PACE helps ensure that figures meet PLOS requirements. To use PACE, you must first register as a user. Registration is free. Then, login and navigate to the UPLOAD tab, where you will find detailed instructions on how to use the tool. If you encounter any issues or have any questions when using PACE, please email PLOS at figures@plos.org. Please note that Supporting Information files do not need this step.

Solution: We made the necessary changes.

3. **Journal Requirements:**

3.1 When submitting your revision, we need you to address these additional requirements.

Please ensure that your manuscript meets PLOS ONE's style requirements, including those for file naming. The PLOS ONE style templates can be found at

https://journals.plos.org/plosone/s/file?id=wjVg/PLOSOne_formatting_sample_main_body.pdf and https://journals.plos.org/plosone/s/file?id=ba62/PLOSOne_formatting_sample_title_authors_affiliations.pdf

Solution: We made the necessary changes.

3.2 Please note that PLOS ONE has specific guidelines on code sharing for submissions in which author-generated code underpins the findings in the manuscript. In these cases, all author-generated code must be made available without restrictions upon publication of the work. Please review our guidelines at https://journals.plos.org/plosone/s/materials-and-software-sharing#loc-sharing-code and ensure that your code is shared in a way that follows best practice and facilitates reproducibility and reuse.

Solution: The code was shared on GitHub, at the link https://github.com/Interfaces-UFSCAR/Codigo-Coleta-PLOS-ONE

3.3 We note that the grant information you provided in the 'Funding Information' and 'Financial Disclosure' sections do not match. When you resubmit, please ensure that you provide the correct grant numbers for the awards you received for your study in the **'Funding Information' section**.

Solution: We made the necessary changes.

3.4 Thank you for stating the following financial disclosure: “This work is supported by FAPESP under grant number 2022/03090-0 under the coordination of the Prof.a Dr.a Sylvia Iasulaitis and BCO number 2023/03704-0 and number 2023/17214-5. This research was funded by the Coordenação de Aperfeiçoamento de Pessoal de Nível Superior - Brasil (CAPES) - Finance Code 001.”

Please state what role the funders took in the study. If the funders had no role, please state: “The funders had no role in study design, data collection and analysis, decision to publish, or preparation of the manuscript.”

If this statement is not correct you must amend it as needed.

Please include this amended Role of Funder statement in your cover letter; we will change the online submission form on your behalf.

Response: “The funders had no involvement in the study design, data collection and analysis, decision to publish, or preparation of the manuscript”. (Cover Letter)

3.5 Thank you for stating the following in the Acknowledgments Section of your manuscript: “This work is supported by FAPESP under grant number 2022/03090-0 under the coordination of the Prof.a Dr.a Sylvia Iasulaitis and BCO number 2023/03704-0 and number 2023/17214-5. This research was funded by the Coordenação de Aperfeiçoamento de Pessoal de Nível Superior - Brasil (CAPES) - Finance Code 001.”

We note that you have provided funding information that is not currently declared in your Funding Statement. However, funding information should not appear in the Acknowledgments section or other areas of your manuscript. We will only publish funding information present in the Funding Statement section of the online submission form.

Please remove any funding-related text from the manuscript and let us know how you would like to update your Funding Statement. Currently, your Funding Statement reads as follows: “This work is supported by FAPESP under grant number 2022/03090-0 under the coordination of the Prof.a Dr.a Sylvia Iasulaitis and BCO number 2023/03704-0 and number 2023/17214-5. This research was funded by the Coordenação de Aperfeiçoamento de Pessoal de Nível Superior - Brasil (CAPES) - Finance Code 001.”

Please include your amended statements within your cover letter; we will change the online submission form on your behalf.

Solution: We made the necessary changes.

This work was funded by FAPESP under grant number 2022/03090-0 under the coordination of Prof. Dr. Sylvia Iasulaitis and BCO number 2023/03704-0 and number 2023/17214-5. This research was supported by the Coordination for the Improvement of Higher Education Personnel - Brazil (CAPES) - Finance Code 001, through the granting of a master’s scholarship. (Cover Letter)

3.6 When completing the data availability statement of the submission form, you indicated that you will make your data available on acceptance. We strongly recommend all authors decide on a data sharing plan before acceptance, as the process can be lengthy and hold up publication timelines. Please note that, though access restrictions are acceptable now, your entire data will need to be made freely accessible if your manuscript is accepted for publication. This policy applies to all data except where public deposition would breach compliance with the protocol approved by your research ethics board. If you are unable to adhere to our open data policy, please kindly revise your statement to explain your reasoning and we will seek the editor's input on an exemption. Please be assured that, once you have provided your new statement, the assessment of your exemption will not hold up the peer review process.

Solution: The data has already been made available on the GitHub account of the Interfaces research group for the purpose of evaluation by the editor and reviewers of PLOS ONE. The access link is https://github.com/Interfaces-UFSCAR/ITED-Br

3.7 Please upload a copy of Supporting Information Figure/Table/etc. Tables 1,2,3,4,5,6,7,8 and Figures 1,2,3,4,5, and 6 which you refer to in your text on pages 24 and 25.

Solution: We made the necessary changes.

**4. Reviewers' comments:**

4.1 Specifically, I'm concerned whether sharing the full text of tweets, along with information about users (Tables 2-5) is in accordance with Twitter's terms of use (ToS) and policies. I know that in the past, public sharing of data was hampered by restrictive license on tweet content. I believe that the authors should outline (either in the article, or as declarations to the editors) what restrictions from the ToS might apply, and how these can be overcome.

Solution: We specified the response in the article in the section "Data Availability." Only tweet ID information will be made available, information from tables 2 to 5 will not be shared publicly.

4.2 The authors state that "All files will be made available on GitHub". Please outline what this entails in more detail (i.e. file format – whether it is the raw download results, or after preprocessing, etc.), the size of the dataset, and any anticipated

issues related to storage (as far as I know, regular Github repositories have storage limitations).

Solution: The data has already been made available on the GitHub account of the Interfaces research group for the purpose of evaluation by the PLOS ONE editor and reviewers, together with the requested description. The access link is https://github.com/Interfaces-UFSCAR/ITED-Br

4.3 The authors could publish the dataset with accompanying documentation in a public repository.

Solution: We made the necessary changes. The access link is: https://github.com/Interfaces-UFSCAR/ITED-Br
