# Supplementary material for: The Interfaces Twitter Elections Dataset: Construction process and characteristics of big social data during the 2022 presidential elections in Brazil
Source: PLoS One. 2025 Feb 3;20(2):e0316626. doi: 10.1371/journal.pone.0316626 (PMC11790155; doi:10.1371/journal.pone.0316626)
Supplement: Attachment — Submitted filename: RESPONSE TO REVIEWERS.pdf [file pone.0316626.s002.pdf]

RESPONSE TO REVIEWERS

Dear Academic Editor
M.D Gábor Vattay, PhD, DSc
PLOS ONE

We would like to express our gratitude to PLOS ONE journal for the opportunity to review the article “The Interfaces Twitter Elections Dataset: construction process and characteristics of Big Social Data during the 2022 presidential elections in Brazil” and for the valuable comments that have helped us to improve our work.

The authors carefully considered each of the reviewers’ and editor’s comments and did their best to address each in a revised version of the article.

After this careful review, we hope the manuscript meets the high standards and requirements of PLOS ONE. Below, we describe the changes made to the text considering each recommendation.

Finally, we clarify that all changes are highlighted in blue in a revised version of the manuscript.

Yours sincerely,

Professor Dr. Sylvia Iasulaitis and authors

1. A clearer response to Reviewer 1's comments on the use of broad terms and false positives- if the authors aren't able to do some validation as suggested, then this may be discussed as a limitation in the Discussion.

Solution: We have included the “Limitations” section in the article.

Although the goal was to limit the amount of data preprocessing to only what was strictly necessary (leaving further refinement for future work), the possibility of conducting a thorough analysis of the presence of false positives in the final dataset was acknowledged. This involves identifying data related to content that does not fit within the sociopolitical scope of the study or does not contribute to the understanding of the initially outlined research objectives. However, due to the scale of the dataset produced, there are numerous and varied methodologies that could be employed to address this issue. Most, if not all, include heuristics that may not yield completely accurate results or rely on subjective definitions, choices, and decisions regarding the attempts to answer questions such as: 'Which Tweets are relevant for studies derived from this dataset?'

The potential benefit of applying such preliminary analysis to future studies based on this work is understood, yet for the reasons highlighted, it is concluded that such data preprocessing falls outside the scope of this study, allowing future work to conduct analyses within their specific contexts and leaving the decision-making power of what is relevant in the hands of those who will use the dataset produced. This conclusion is further reinforced when considering that most studies will likely use a subset of the dataset or data pertaining to a specific context (political, for example). In many cases, identifying false positives, given the broad range of potential contexts, may require considering many subtleties. These subtleties and the presence of multiple contexts justify the presence of different methodologies for selection - of which, probably the most appropriate are AI based - and which, to achieve the best results, would require a different input, training data, or fine-tuning, depending on the target context.

Finally, despite this limitation, confidence in the value of this dataset remains, given the scarcity of large datasets like this available in the academic-scientific context; and also in light of the considerations and decisions made by specialists in sociopolitical studies, which led to the preliminary observations and the construction of the queries used for data collection, as outlined in this study. We believe that future users of this dataset will be able to extract that value using existing and well-known methodologies for filtering text-based datasets chosen based on the specific objectives and/or political context that they choose to study.

2. Additional supporting references, particularly in the Discussion. Specifically, this section contains statements that are not directly supported by the results or

other citations. For example, the authors say "Negative propaganda against opponents, a resource commonly employed in electoral disputes, has played a prominent role in Jair Bolsonaro's campaigns since the 2018 Brazilian presidential election." This would need a supporting reference or removal.

Solution: This has been removed.

3. In addition to the above point, the authors state "In such scenarios, the potential for negative propaganda through disinformation arises, as the connection between official coalitions or party federations (from 2022) and unofficial groups responsible for disseminating campaign content cannot be easily proven [32], making it challenging to penalize those involved." This seems to be saying that they can't confirm the campaign members were directly involved. As such, we request that the authors simply report objectively what happened here, rather than framing it speculatively as is.

Solution: This has been removed.

4. Clarification on what the date range of published tweets that were included in the study are.

Response:
The period of analysis of the data presented in the article can be found on page 20 in the section "Analysis of the sample," namely:

The sample analysis was based on a temporal cut of the database, covering the period from October 27, 2022, to November 02, 2022. This time frame encompasses the pre-election days (October 27, 28, 29, and 30) and the post-election days (October 31, November 01, and 02).

5. **Fig 5 appears to be copyrighted, and we require clarification on the appropriate permissions for this image.**

Solution: Inserted the source in the image description in the original article: Source: instagram.com/tiago_asmar and facebook.com/cirogomesoficial

Response: Considering Brazilian legislation, according to Law No. 9,610 of February 19, 1998, Chapter IV, Article 46, the reproduction of certain copyrighted material (of which classification the subject images fall under) in scientific works does not constitute

a violation of copyright (available at http://www.planalto.gov.br/Ccivil_03/leis/L9610.htm). Regarding U.S. legislation, the Fair Use policy available on the X platform itself (https://help.x.com/en/rules-and-policies/fair-use-policy) considers the image as "Transformative for educational and non-commercial use."

In addition to what applies under the Fair Use policy, summarizing what is relevant regarding image copyright on X (formerly Twitter) – content (object: Tweet, User, Media, etc.) extracted from the platform via the API, under terms applicable to the researchers at the time of data collection, may be distributed in its entirety at a volume of 50,000 per day per consumer. In this case (pertaining to the to-be-published article), two objects (2 images) are shared with each article reader. The remainder of the dataset, which will be shared via GitHub, is not included in this count, as it contains only the IDs of each object, which, according to the same terms, may be shared without restrictions in terms of quantity or volume.

Regarding creative authorship, efforts were made to identify the authorship of the two images that make up Figure 5 (A and B) through backtracking with search platforms. Two social media profiles were found (Instagram for Figure 5A; Facebook for Figure 5B), with high confidence that these are the original authors of the images used. This is corroborated by the fact that both images, in at least one instance each, are credited to the aforementioned authors by well-regarded news outlets in Brazil.

For the Facebook and Instagram platforms (where the images were originally identified), Meta's Fair Use rules apply, similar to X (available at https://www.meta.com/help/policies/meta-policies/meta-copyright/).

To emphasize, it is believed, again with high confidence, that under U.S. law, the intended use of these images can be classified as Fair Use, as they are: 1- used for scientific purposes; 2- of low creative value (both are simple edits of Brazilian political figures); and 3- used in a way that will not impact their monetization (if they are monetized).

Furthermore, the original profiles will be credited under each image for ethical purposes, and, at the end of the text, a short paragraph was added clarifying the situation regarding the copyright of the images, stating: "The images from Figure 5 (A and B) are included under Fair Use for scholarly purposes. All rights to the original images remain with their respective copyright holders."
